# The protective effect of endurance running against the pro-invasive effects of ageing in breast cancer cells and mesenchymal stem cells *in vitro*

**DOI:** 10.1007/s44164-023-00055-y

**Published:** 2023-08-02

**Authors:** Marie-Juliet Brown, Matt Nickels, Elizabeth C. Akam, Mhairi A. Morris

**Affiliations:** School of Sport, Exercise and Health Sciences, Loughborough University, Towers Way, Loughborough, LE11 3TU UK

**Keywords:** Breast cancer cells, Mesenchymal stem cells, Invasion, Exercised serum, Endurance running, Sprinting

## Abstract

**Purpose:**

Regular exercise is known to reduce cancer risk and may prevent metastases, however, modelling this *in vitro* is challenging due the heterogeneity of the tumour microenvironment. Exercised serum can be used to capture changes in cellular signalling components in response to different types and durations of exercise. In this study, exercised serum from long-term endurance runners and sprinters of different ages was used to evaluate the impact of exercise on the invasiveness of breast cancer cells and mesenchymal stem cells *in vitro*.

**Methods:**

Exercised serum from long-term trained younger and older endurance runners and sprinters was used to supplement cell culture media in the 3D culture of spheroids containing breast cancer cells or mesenchymal stem cells. Spheroids were generated in a 3D semi-solid matrix and cell invasion was measured using ImageJ software. Statistical analyses of invasion were conducted using one-way ANOVAs.

**Results:**

Invasion was significantly greater in cells cultured with serum from older, inactive participants compared to young, inactive participants (YC vs OC; F _(1,3)_ = 37.135, *P* = 0.009). No significant difference was found in the invasion of MDA-MB-231 breast cancer cells cultured in serum from older, long-term endurance runners and younger, long-term endurance runners (YE vs OE; F _(1,3)_ = 5.178, *P* = 0.107), suggesting a protective effect of endurance running against the pro-invasive effects of ageing.

**Conclusion:**

This is the first study of its kind to demonstrate the protective effects of long-term exercise training type in two populations of different ages against the invasiveness of breast cancer cells *in vitro*.

## Introduction

Breast cancer is the most frequently diagnosed malignancy and the second leading cause of cancer-related mortality in women worldwide [1, 2]. The number of newly diagnosed breast cancer cases in 2020 was approximately 2.3 million [1] and this number is expected to continue to increase. Despite improvements in survival rates, metastatic breast cancer continues to be fatal within 5–10 years [3] and 20–30% of patients with early breast cancer die of metastatic disease [4].

Mesenchymal stem cells (MSCs) are multi-potent stromal cells that exist within the tumour microenvironment (TME), and are heavily implicated in tumour growth, metastasis, and progression [5–8]. Human bone marrow-derived MSCs (hBM-MSCs) are known to home towards tumours with high affinity, due to the inflammatory nature of tumours [9]. Once there, they incorporate themselves into the tumour itself [10], where they subsequently communicate with the surrounding tumour and stromal cells and can exert both pro- and anti-tumourigenic effects [5, 7, 11–13]. As a result, MSCs are potentially a critical point of research in the TME and progression of breast cancer.

Traditionally, *in vitro* cancer research has been conducted using cells cultured in two dimensions, and most of the data on cancer progression is derived from experiments in conventional monolayer cultures (2D culture). However, it is now more widely understood that 2D models fail to represent the tumour microenvironment in a physiologically relevant manner. The characteristics of cells cultured in 2D and 3D are different; 2D cells can lose their epithelial cell polarity, display different patterns of gene expression, show altered cellular communication and morphological changes all of which affect their function [14–16]. The unlimited access to cell culture medium for cancer cells cultured in 2D is also unrepresentative of the environment found i*n vivo*; the availability of oxygen and other nutrients is much more varied *in vivo* due to the structure of tumours naturally [17]. Subsequently, in recent years, there has been a steady transition from the use of 2D models to 3D models to study cancer *in vitro*. These 3D models can more closely mimic the environment found *in vivo* and therefore provide results that are far more indicative of *in vivo* responses than prior 2D models.

A wealth of evidence supports a strong association between partaking in regular exercise or physical activity and a decreased risk of developing cancer [18, 19]. The international Agency for Research on Cancer of the World Health Organisation estimate a 20% to 40% decrease in risk of developing breast cancer in women who are most physically active [20]. There is also evidence that shows increasing physical activity levels once diagnosed with breast cancer can reduce breast cancer mortality risk [21, 22]. However, the body of evidence that investigates the effects of prolonged exercise training (“chronic exercise”) on breast cancer cell invasion *in vitro*, is extremely small. Cross-sectional studies have shown lower prostate cancer cell growth and higher degrees of apoptosis when cultured with sera from regular exercisers compared to inactive control participants [23–25] Some studies have investigated the effects of exercised serum on breast cancer cell lines, but these have either involved short-term acute exercise interventions (4 -9 weeks) [26, 27] or longer (6 month) training interventions [28].

Recently Metcalfe et al. (2021) identified key research gaps on exercise-conditioned serum and cancer cell behaviour and confirmed that there is limited research on the effect that training type and duration has on cancer progression *in vitro* [29]*.* Therefore, the aim of this study is to determine the effects of age and long-term exercise training type (defined herein as 11 + years of regular exercise) on the invasion of breast cancer cells or stromal hBM-MSCs *in vitro*, since previous studies have shown that altering the invasiveness of hBM-MSCs may contribute to reduced breast cancer progression [30]. Findings presented herein support the idea that long-term exercise training has a protective effect against breast cancer cell invasion *in vitro*.

## Methods

### Participants

Briefly, serum from 9 young control (YC) participants, 10 young sprinters (YS), 11 young endurance (YE) runners and 7 older controls (OC), 9 older sprinters (OS) and 11 older endurance (OE) runners was used in this study. Participants were recruited via word of mouth and internet advertisements. All participants were required to complete the Warwick-Edinburgh Mental Wellbeing Scale to assess their health and mental wellbeing status. Inclusion criteria were as follows: no known metabolic disorders, cardiovascular disease or cancer; non-smokers; no use of anti-inflammatory medication or steroids; and free of any condition that could influence outcome measurements. The recruitment of participants to the original study was approved by the Ethics Review Subcommittee at Loughborough University (R19-P056) and complied with the 1964 Declaration of Helsinki. Informed consent was given by all participants before data collection.

### Athletic categorisation

Age groupings were determined according to the convention established in athletics of “master athletes” being those aged ≥ 35 years [31]. ‘Young’ athletes were 18–34 years old, and ‘older’ athletes were 35–60 years old [32]. All athletes possessed a minimum of 5 years of regular competitive practice. Athletes were considered sprinters if they competed in events 60–400 m and endurance runners if they competed in distances > 3,000 m.

All young (18–34 years) and older controls (35–60 years) were untrained and had no engagement in any routine exercise (for details refer to Table 1).

**Table 1:** Body composition of athletes and age-matched controls

	YC	YS	YE	OC	OS	OE
*n*	9 (5 m, 4f)	10 (6 m, 4f)	11 (6 m, 5f)	7 (4 m, 3f)	9 (4 m,5f)	11 (6 m, 5f)
Age (yrs)	24.3 (± 1.32)	23.8 (± 4.21)	25.45 (± 3.88)	52.59 (± 7.91)	51.00 (± 11.26)	45.82 (± 5.06)
Height (cm)	171.7 (± 9.96)	175.62 (± 8.62)	175.75 (± 8.82)	169.17 (± 11.45)	174.27 (± 8.51)	170.05 (± 9.79)
Body mass (kg)	67.4 (± 9.29)	70.07 (± 12.48)	61.68 (± 9.05)	86.49 (± 16.57)	73.72 (± 12.81)	63.51 (± 8.65)
BMI (kg/m^2^)	24.3 (± 1.32)	22.54 (± 2.06)	19.84 (± 4.79)	30.07 (± 3.83)	24.12 (± 2.68)	21.85 (± 1.84)
Body fat (%)	19.2 (± 8.90)	13.26 (± 1.94)	12.93 (± 4.79)	33.30 (± 6.30)	20.43 (± 4.71)	15.65 (± 5.18)
FFM (kg)	54.6 (± 10.48)	60.88 (± 11.49)	53.92 (± 9.48)	58.01 (± 14.28)	58.72 (± 11.11)	53.80 (± 9.47)

Means ± SD. *M* Male; *F* Female; *BMI* Body mass index; *FFM* Fat free mass; *YC* Young control; *YS* Young sprinters; *YE* Young endurance; *OC* Older control; *OS* Older sprinters; *OE* Older endurance

Young sprint athletes had an average of 11.2 ± 4.0 years of training or competitive practice, spent an average of 11.7 ± 0.6 h training per week (average three sprint and two weight training sessions), and had a maximal time of inactivity of 4.4 ± 3.8 months. Older sprinters had an average of 31.7 ± 14.6 years of training or competitive practice, spent an average of 6.8 ± 2.3 h training per week (average three sprint and two weight training sessions) and had a maximal time of inactivity of 11.4 ± 12.4 months.

The YE athletes had an average of 11.6 ± 7.0 years of training or competitive practice, covered an average training distance of 49.1 ± 19.1 miles per week, spent an average of 8.0 ± 3.0 h training per week, and had a maximal time of inactivity (e.g., injury) of 6.7 ± 8.9 months. OE had an average of 20.45 ± 13.3 years of training or competitive practice, covered an average training distance of 40.2 ± 16.9 miles per week, spent an average of 6.7 ± 2.4 h training per week, and had a maximal time of inactivity of 11.0 ± 14.8 months.

### Sample collection and processing

Anticoagulant-free vacutainers (Becton, Dickson & Company, UK) were used for serum.isolation. A trained phlebotomist collected blood samples from participants. These samples were kept at room temperature for 30–60 min prior to centrifugation at 1400xg (4100 rpm) for 5 min (20 °C). The collected supernatant from the centrifuged blood samples was recentrifuged using the same conditions and to ensure the complete removal of any residual cells. Serum was removed and aliquots were stored at -80 oC until required.

### Cell culture

The following cell lines were used: T47D (human, ductal carcinoma of the breast; ECACC 85102201) and MDA-MB-231 (human Caucasian breast adenocarcinoma, invasive; ECACC 92020424). Both cancer cell lines were supplied by the European Collection of Authenticated Cell Cultures, and normal human bone marrow-derived mesenchymal stem cells (hBM-MSCs) were supplied by Lonza Poietics ™, USA (Catalogue number: PT-2501; Lot number: 071313B). Cells were cultured in Dulbecco’s Modified Eagle Medium (DMEM; Gibco ™, Thermo Fisher, UK) low glucose (1 g/L) + GlutaMAX supplemented with 10% foetal bovine serum (FBS) from Rocky Mountain Biologicals, in Corning® T175 or T75 flasks and incubated until confluent at 37.5 °C and 5% CO_2_. Media was replaced every 3 days.

### Generation of spheroids

Wells of a 96-well plate were rinsed in anti-adherence rinsing solution (AARS; STEMCELL Technologies, UK) before seeding 4500 cells/well in a working volume of 100mL of media per well. Cells were incubated at 37.5 °C and 5% CO_2_ and left to form spheroids for 3–4 days.

### Invasion assays

Spheroids were embedded into a matrix made up of growth-factor reduced base membrane extract (BME; 8–12 mg/mL; Bio-Techne Ltd., UK) and rat tail collagen type 1 at 5 mg/mL, at a 1:1 ratio (R&D Systems, USA). BME was thawed on ice and kept on ice along with rat collagen type I. Sufficient volumes of each reagent required to embed the correct number of spheroids (e.g., 150mL of BME, mixed with 150mL rat collagen type I to create enough matrix to embed 10 spheroids) were collected into pre-chilled 0.5 mL tubes. Then, 30mL of the BME/Collagen matrix was added into the centre of a single well of a 24-well plate. Finally, a spheroid was gently placed into the matrix using a 20mL pipette. The matrix was left in an incubator at 37.5 °C and 5% CO_2_. This procedure was repeated to obtain triplicates of spheroid-containing wells. Spheroids were left for 5 days embedded in the matrix with media replaced every 24 h. Images were taken daily.

### Imaging

The invasion of each spheroid was observed by obtaining daily images taken within the same period (± 15 min) using an inverted laboratory Leica microscope, a 4 × objective with attached Leica MC170 HD camera, and Leica Application Suite (LAS) software (Leica Microsystems, UK).

### Analysis of images

Invasion was calculated using the same methods reported in Brown et al., 2019 [33]. Briefly, invasion was analysed using the multi-point tool on ImageJ. The co-ordinates of the cellular core centre were measured and then co-ordinates for the outer most invaded cells (perimeter of invaded area) were collected. The linear distance of each invaded cell from the centre of the core was then calculated.

### Statistical analyses

All analyses were conducted using IBM SPSS Statistics 27 (IBM Corporation, USA). Data was tested for normal distribution. Normally distributed data were analysed using one-way ANOVAs and a repeated measures generalised linear model to determine main effects.

## Results

### Effect of chronic exercise type on invasion

#### MDA-MB-231 invasion with young-trained serum

**Fig. 1:**
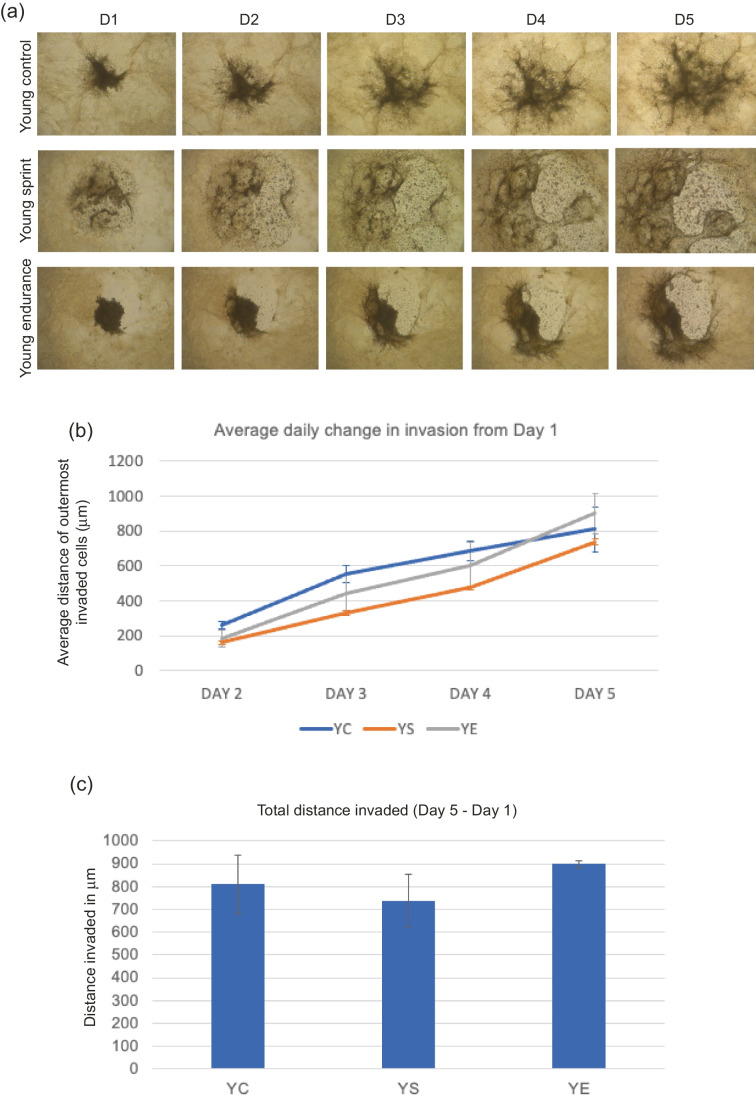
**a** Representative images of MDA-MB-231 spheroid invasion in a BME/rat collagen Type I matrix from Day 1 to Day 5 in YOUNG control, sprint and endurance exercised serum; **(b)** average distance (mm) of outermost invaded cells was measured using the multi-point tool on ImageJ, and data were analysed using one-way ANOVAs and a repeated measures generalized linear model to determine main effects; **(c)** Total distance invaded was measured by subtracting the distance invaded by day 1 from the distance invaded by day 5. No significant main effect of condition was observed (*P* = 0.339) and the reduction in total invasion in spheroids cultured in young serum was also not significant (*P* = 0.667). Data representative of biological triplicates with standard error bars presented. Abbreviations: YC = young control; YE = young endurance; YS = young sprint

Total distance invaded was calculated by subtracting Day 1 distance from Day 5 distance. No significant main effect of condition (F _(1,5)_ = 1.354, *P* = 0.339) was found, where average daily change was lowest in YS across all days (Fig. 1). Total invasion appeared to reduce in YS conditions and increase in YE conditions compared to control, but these results were not significant (*P* = 0.0667, mean total distance invaded; YC—809.63 μm, YS – 737.27 μm, YE – 899.48 μm).

#### MDA-MB-231 invasion with older-trained serum

**Fig. 2:**
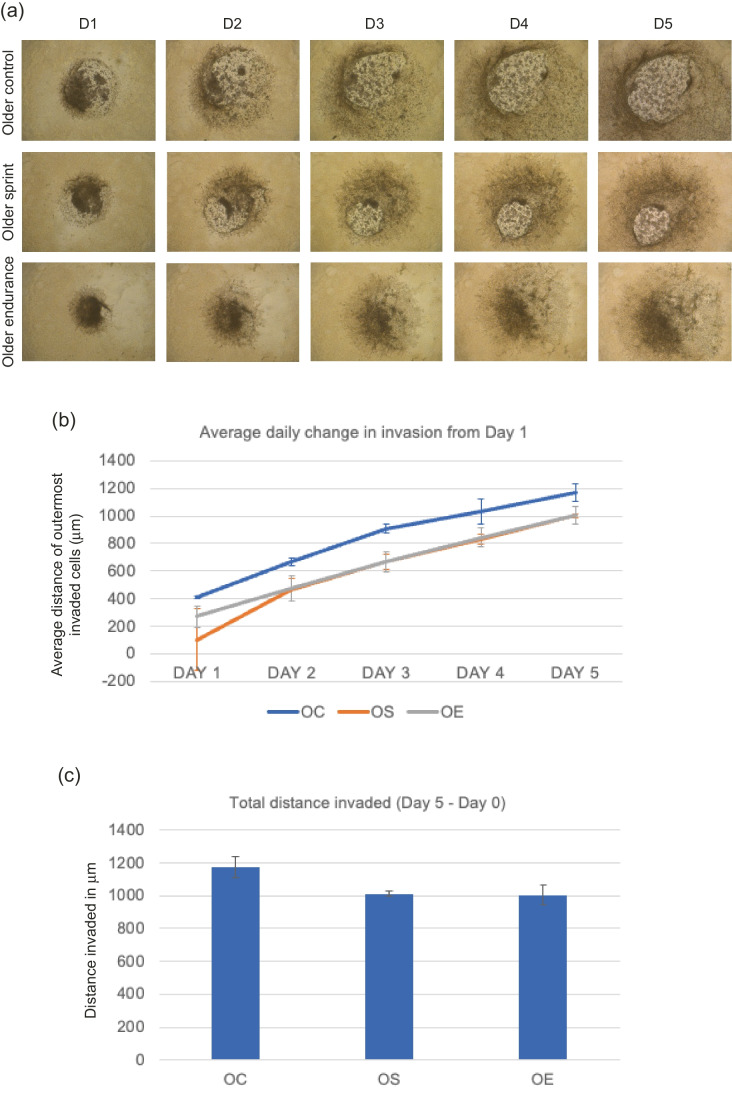
**a** Representative images of MDA-MB-231 spheroid invasion in a BME/rat collagen Type I matrix from Day 1 to Day 5 in OLDER control, sprint and endurance exercised serum; **(b)** average distance (mm) of outermost invaded cells was measured using the multi-point tool on ImageJ, and data were analysed using one-way ANOVAs and a repeated measures generalized linear model to determine main effects; **(c)** Total distance invaded was measured by subtracting the distance invaded by day 1 from the distance invaded by day 5. No significant main effect of condition was observed (*P* = 0.219) and the reduction in total invasion in spheroids cultured in older serum was also not significant (*P* = 0.139). Data representative of biological triplicates with standard error bars presented. Abbreviations: OC = older control; OE = older endurance; OS = older sprint

No significant main effect of condition was found (OC, OS or OE; F _(1,5)_ = 2.087, *P* = 0.219) where daily change across all days was greatest in the older control condition (Fig. 2). Total invasion reduced in sprint and endurance conditions compared to the control, but these changes were not statistically significant (*P* = 0.139, mean total distance invaded for OC- 1172.51 μm, OS- 1012.95 μm, OE- 1005.96 μm).

#### T47D invasion with young-trained serum

**Fig. 3:**
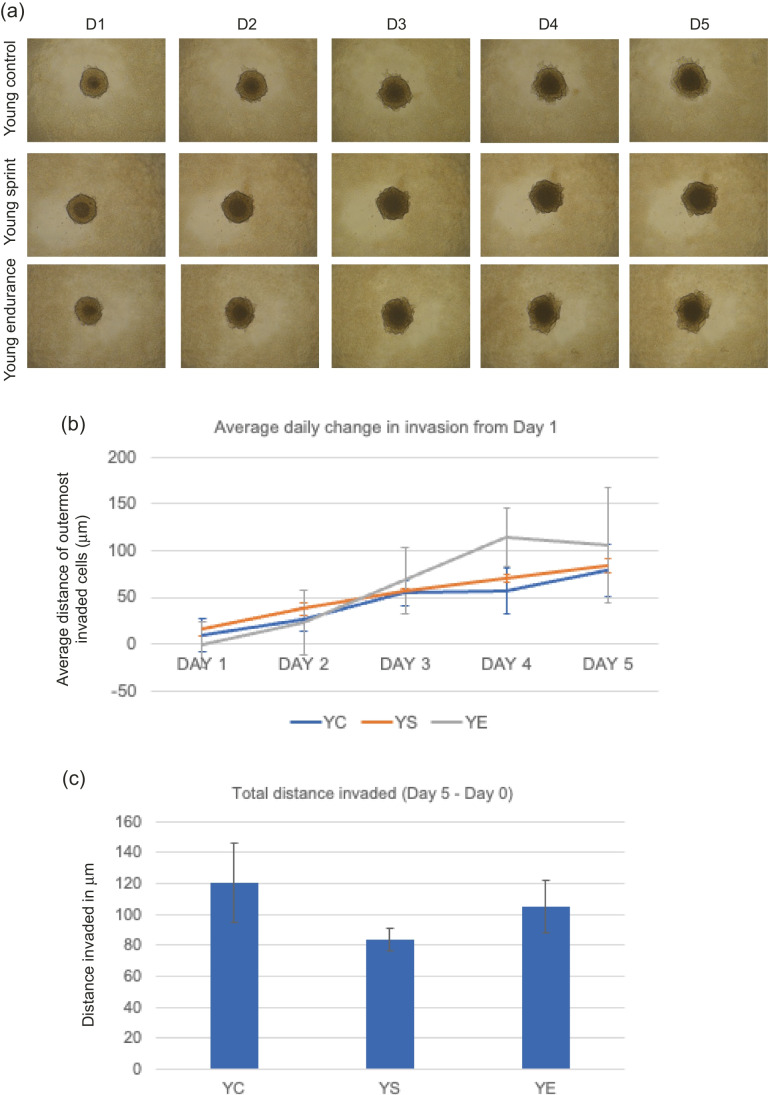
**a** Representative images of T47D spheroid invasion in a BME/rat collagen Type I matrix from Day 1 to Day 5 in YOUNG control, sprint and endurance exercised serum; **(b)** average distance (mm) of outermost invaded cells was measured using the multi-point tool on ImageJ, and data were analysed using one-way ANOVAs and a repeated measures generalized linear model to determine main effects; **(c)** Total distance invaded was measured by subtracting the distance invaded by day 1 from the distance invaded by day 5. No significant main effect of condition was observed (*P* = 0.878) and the reduction in total invasion in spheroids cultured in young serum was also not significant (*P* = 0.801). Data representative of biological triplicates with standard error bars presented. Abbreviations: YC = young control; YE = young endurance; YS = young sprint

No significant main effect of condition (YC, YS or YE; F _(1,6)_ = 0.132, *P* = 0.878) was found, where average daily change across all days was greatest in YE (Fig. 3). Total invasion appeared to be lowest in YS conditions, but these changes were not statistically significant (*P* = 0.801, YC – 120.50 μm, YS – 83.72 μm, YE – 105.41 μm).

#### T47D invasion with older-trained serum

**Fig. 4:**
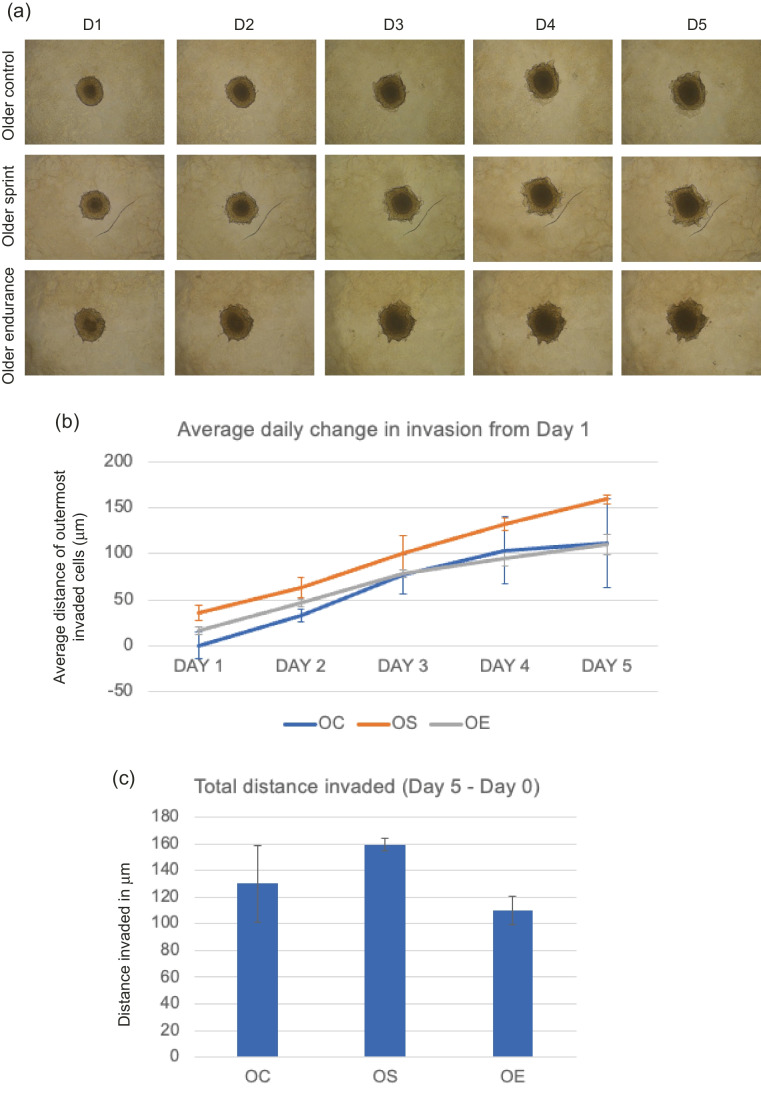
**a** Representative images of T47D spheroid invasion in a BME/rat collagen Type I matrix from Day 1 to Day 5 in OLDER control, sprint and endurance exercised serum; **(b)** average distance (mm) of outermost invaded cells was measured using the multi-point tool on ImageJ, and data were analysed using one-way ANOVAs and a repeated measures generalized linear model to determine main effects; **(c)** Total distance invaded was measured by subtracting the distance invaded by day 1 from the distance invaded by day 5. No significant main effect of condition was observed (*P* = 0.596) and the reduction in total invasion in spheroids cultured in older serum was also not significant (*P* = 0.706). Data representative of biological triplicates with standard error bars presented. Abbreviations: OC = older control; OE = older endurance; OS = older sprint

Average daily change in invasion appeared to be greatest in OS compared to control and endurance (Fig. 4), but no significant differences were found in the main effect of condition (OC, OS or OE; F _(1,5)_ = 0.574, *P* = 0.596). There were no significant differences found between total distance invaded across groups (*P* = 0.231), where OS total invasion was greater than the control, and endurance serum appeared to reduce total invasion.

#### hBM-MSC invasion with young-trained serum

**Fig. 5:**
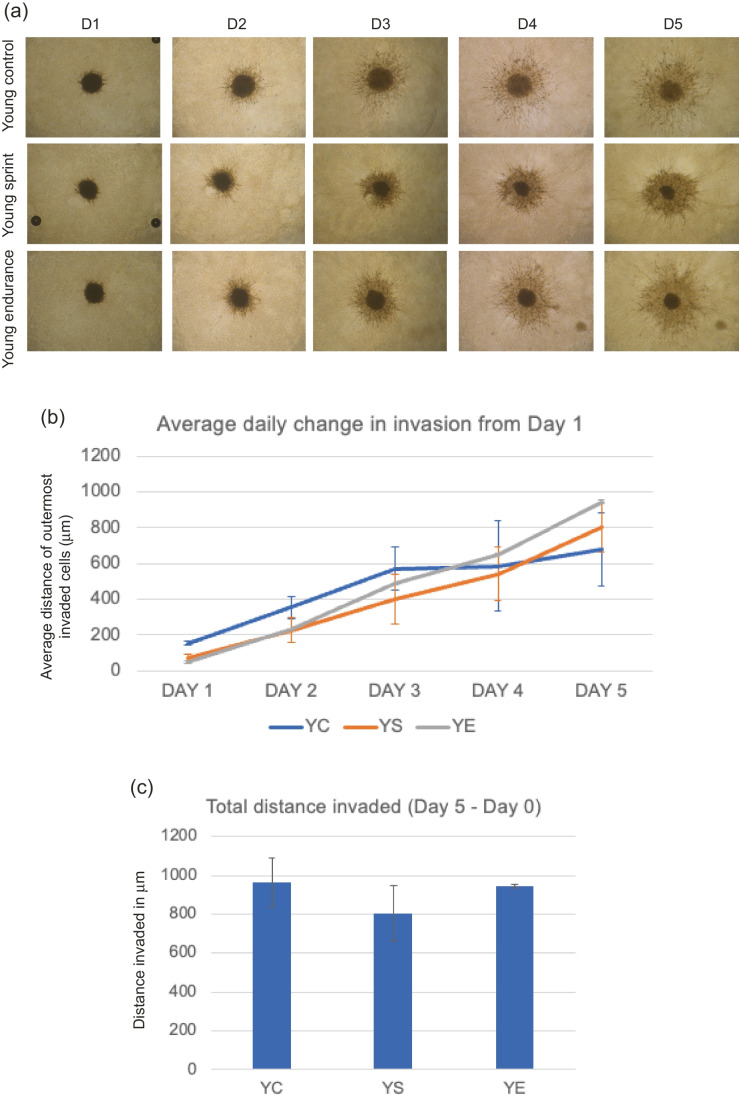
**a** Representative images of hBM-MSC spheroid invasion in a BME/rat collagen Type I matrix from Day 1 to Day 5 in YOUNG control, sprint and endurance exercised serum; **(b)** average distance (mm) of outermost invaded cells was measured using the multi-point tool on ImageJ, and data were analysed using one-way ANOVAs and a repeated measures generalized linear model to determine main effects; **(c)** Total distance invaded was measured by subtracting the distance invaded by day 1 from the distance invaded by day 5. The distance invaded by spheroids cultured in YC serum on Day 1 was significantly greater than those cultured in YS serum (*P* = 0.018) and YE serum (*P* = 0.011). No significant main effect of condition was observed (*P* = 0.892) and the reduction in total invasion in spheroids cultured in young serum was also not significant (*P* = 0.635). Data representative of biological triplicates with standard error bars presented. Abbreviations: YC = young control; YE = young endurance; YS = young sprint

No significant main effect of condition (YC, YS or YE; F _(1,5)_ = 0.117, *P* = 0.892) was found, where average daily change across all days was greatest in YC (Fig. 5). Total invasion was lower in YS condition compared to control and endurance, but these differences were not significant (*P* = 0.635; YC-962.69 μm, YS- 805.08 μm, YE – 945.49 μm).

#### hBM-MSC invasion with older-trained serum

**Fig. 6:**
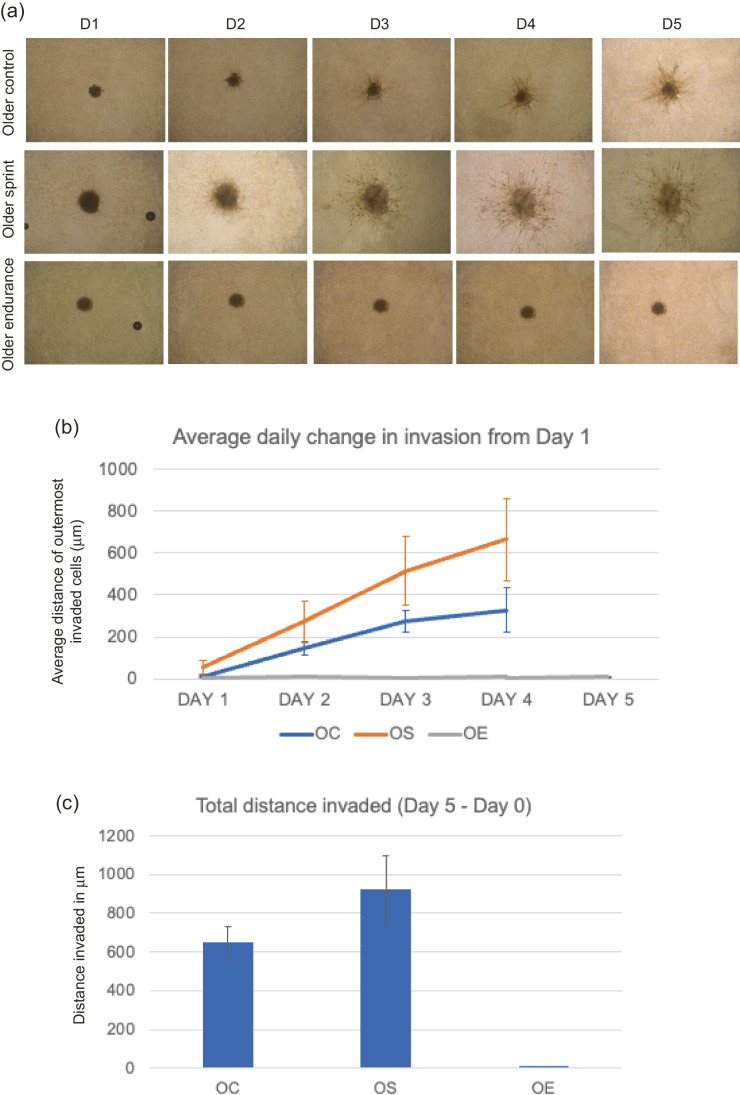
**a** Representative images of hBM-MSC spheroid invasion in a BME/rat collagen Type I matrix from Day 1 to Day 5 in OLDER control, sprint and endurance exercised serum; **(b)** average distance (mm) of outermost invaded cells was measured using the multi-point tool on ImageJ, and data were analysed using one-way ANOVAs and a repeated measures generalized linear model to determine main effects; **(c)** Total distance invaded was measured by subtracting the distance invaded by day 1 from the distance invaded by day 5. A significant main effect of condition was observed (*P* = 0.020) and the reduction in total invasion in spheroids cultured in older serum was also not significant (*P* = 0.003). Data representative of biological triplicates with standard error bars presented. Abbreviations: OC = older control; OE = older endurance; OS = older sprint

A significant main effect of condition (OC, OS or OE; F _(1,6)_ = 8.076, *P* = 0.020) was found, where average daily change across all days was lowest in OE spheroids (Fig. 6). A significant difference in total invasion was found where total distance invaded was significantly lower in OE compared to control (648.307 vs 9.607, *P* = 0.007).

### Effect of age on invasion

#### MDA-MB-231 invasion with control serum

A significant main effect of condition (YC vs OC; F _(1,3)_ = 37.135, *P* = 0.009) was found, where average daily change across all days was significantly lower in YC compared to OC, suggesting serum from older participants who were not trained led to significant increased invasion compared to young control participants.

#### MDA-MB-231 invasion with sprinters’ serum

A significant main effect of age (YS vs OS; F _(1,3)_ = 7.951, *P* = 0.048) was found, where average daily change across all days was significantly lower in YS compared to OS, suggesting serum from older sprinters significantly increases invasion risk compared to young sprinters.

#### MDA-MB-231 invasion with endurance runner serum

No significant main effect of condition (YE vs OE; F _(1,3)_ = 5.178, *P* = 0.107) was found, where average daily change across all days was lower in YE compared to OE but this was not significant, suggesting no significant difference in invasion occurred between serum from young vs older endurance runners.

#### T47D invasion with control serum

No significant main effect of condition (YC vs OC; F _(1,4)_ = 0.476, *P* = 0.528) was found, where average daily change across all days was lower in YC compared to OC but this was not significant.

#### T47D invasion with sprinters’ serum

Significant main effect of condition (YS vs OS; F _(1,3)_ = 12.390, *P* = 0.039) was found, where average daily change across all days was significantly lower in YS compared to OS (52.852 vs 92.189 respectively). A significant difference between YS and OS in total distance invaded (F _(1,5)_ = 81.203, *P* < 0.001) was also found, where YS invasion was lower than in OS (83.723 vs 159.256 respectively).

#### T47D invasion with endurance runner serum

No significant main effect of condition (YE vs OE; F _(1,4)_ = 0.036, *P* = 0.858) was found, where average daily change across all days was lower in YE compared to OE but this was not significant (61.936 vs 69.075 respectively).

#### hBM-MSC invasion with control serum

No significant main effect of condition (YC vs OC; F _(1,4)_ = 2.629, *P* = 0.180) was found, where average daily change across all days was lower in OC compared to YC but this was not statistically significant.

#### hBM-MSC invasion with sprinters’ serum

No significant main effect of condition (YS vs OS; F _(1,4)_ = 0.218, *P* = 0.665) was found, where average daily change across all days was lower in YS compared to OS (408.151 vs 486.162 respectively).

#### hBM-MSC invasion with endurance runner serum

A significant main effect of condition (YE vs OE; F _(1,3)_ = 34,631.946, *P* < 0.001) was found, where average daily change across all days was much greater in YE compared to OE (471.550 vs 7.930 respectively). A significant difference between YE and OE in total distance invaded (F _(1,4)_ = 13703.239, *P* < 0.001) was also found, where the YE mean was 945.4950 and OE mean was 9.6071. A summary of the effects of exercised serum from younger and older runners on the invasion of spheroids in each cell type is given in Tables 2 and 3.

**Table 2:**
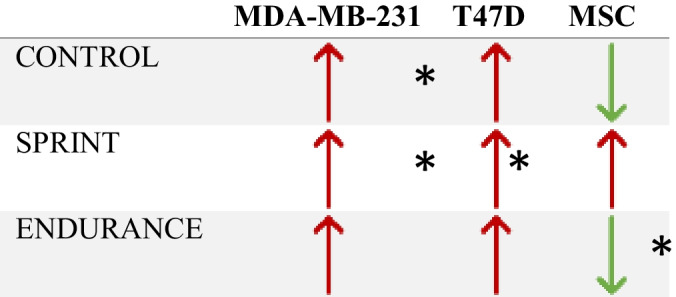
Summary of results – comparison of invasion of cells with serum from different aged participants within the same exercise groups

Upwards arrow indicates greater invasion in older group vs young. Downwards arrow indicates lower invasion in older group vs young

*indicates statistically significant differences observed between older and young groups

**Table 3:**
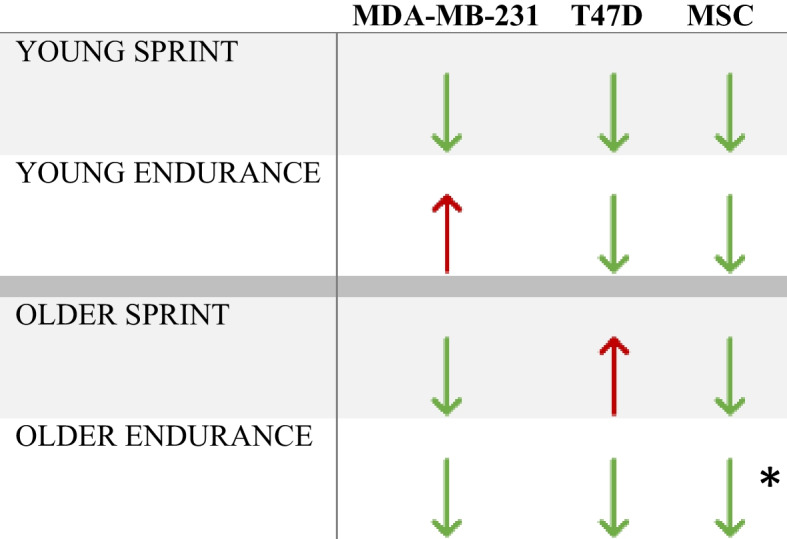
Summary of results – comparison of invasion of cells with serum from different exercise groups within the same age groups compared to respective controls

Upwards arrow indicates greater invasion in exercise group compared to control. Downwards arrow indicates lower invasion in exercise group compared to control

*indicates statistically significant differences observed between exercise and control group

## Discussion

The data presented in this study demonstrates that chronic training has the potential to reduce levels of both breast cancer cell and mesenchymal stem cell invasion *in vitro*. No significant difference was determined between the effects of sera from chronically trained individuals of the same age (endurance runners or sprinters) on breast cancer cell invasion *in vitro* compared to untrained controls. Statistically different levels of invasion in MDA-MB-231 cells were observed when cultured in sera from exercise-matched participants of different ages: sera from young control participants showed reduced MDA-MB-231 cell invasion when compared with sera from older control participants, and sera from young sprint athletes also showed reduced MDA-MB-231 cell invasion when compared with sera from older sprint athletes, supporting the current body of evidence that age plays a significant role in metastasis *in vivo* [34].

Numerous studies have shown that levels of several inflammatory cytokines, including IL-6, IL-8, C-reactive protein (CRP) and TNFα increase with age, even in healthy individuals [35–37] This progressive tendency towards a pro-inflammatory phenotype with age was coined “inflammageing” [38] and plays a role in many age-related diseases, including cancer [35, 39, 40]. Consequently, the augmented basal level of inflammation associated with older age results in numerous factors that could increase the likelihood of a poorer prognosis in breast cancer. The findings presented in this study could potentially be explained by these age-related factors and thus warrant further investigation.

Critically, no significant difference was observed between the invasion of MDA-MB-231 cells cultured with serum from young and older endurance athletes (YE vs OE; F _(1,3)_ = 5.178, *P* = 0.107), suggesting long-term participation in endurance running could prevent the increase in breast cancer cell invasion observed with age. When the levels of inflammatory markers such as TNFα and IL-8 in the exercised serum from participants included in our study were measured, there were no significant differences in concentrations between groups [41]. However, the OC group did have higher levels of CRP than OE and OS (*P* < 0.01), suggesting that chronic exercise training can reduce the age-associated increases in CRP. This is a noteworthy finding that suggests chronic endurance training may have protective effects against the naturally observed increased levels of inflammatory markers with ageing, however, further studies must be conducted to confirm this. These results are also supported by literature that reports increases in CRP levels with ageing [42]. Other studies have found that older, life-long endurance runners had lower CRP and IL-6 markers (*p* < 0.05) compared to age-matched, untrained controls [43], again demonstrating the effect of long-term endurance running on systemic inflammation. These findings support the notion that chronic endurance training could positively alter breast cancer progression.

The results of the current study revealed a statistically significant difference in invasion of MDA-MB-231 cells when cultured in serum from young vs older controls, and young vs older sprinters, where both young groups had lower invasion when compared to older, exercise-matched groups. In the current body of literature, inconsistent findings regarding the effects of sprint training on inflammatory markers are reported: one study showed no difference in inflammatory markers between groups who participated in sprint training vs high-intensity interval training (HIIT) [44] whilst another reported increased levels of IL-6 and TNFα after sprint training in a small sample of young men and women [45]. It is important to note that these discrepancies could be due to differences in the methods and populations (e.g., age, health status) involved in the studies, since one study investigated the effects of sprint training in a sedentary population, whilst the other study’s participant profile was at least minimally active, and had higher baseline fitness levels to begin with [44].

Many studies that report a positive effect of exercise involve participants who complete only a single bout of exercise and investigate the differences between baseline measures and measures immediately after an acute exercise intervention (i.e., cross-sectional study design). It is therefore difficult to determine whether any of these observed positive effects are sustained longer term. As such, it is important to understand whether changes in inflammatory markers that occur after a single bout of exercise are maintained after regular exercise, such as in ‘trained’ individuals.

One previous study investigated the effect of four days of moderate intensity exercise on cytokine levels in healthy men and women [46]. The authors found that concentrations of IL-6, IL-10 and IL-8 all increased immediately after exercise from baseline, and then declined from day 1 to day 2 (*p* < 0.01), remaining stable thereafter. Another study found that individuals who repeated the exact same cycling exercise after 1 week, had an attenuated cytokine response [47]. This suggests that individuals rapidly adapt to repeated exercise and therefore the effect of regular exercise training may attenuate both the pro- and anti-inflammatory responses seen after acute exercise. In a similar vein, the anti-invasive effects of endurance exercise against age-related invasiveness in breast cancer cells observed in the current study may not be solely attributable to the changes in inflammatory markers after individual exercise bouts completed by participants, but by their overall inflammatory status, which may well be mediated by other factors, such as levels of body fat resulting from exercise, or sex hormones [48–50].

Additionally, the half-life of these inflammatory markers in serum is around 19 h for CRP [51], but the half-life of IL-8 and TNFa are much shorter: 24 min and 18.2 min, respectively [52]. *In vivo,* cells are exposed to serum profiles that are changing continuously, rather than simply for a fixed and/or singular moment. *In vitro* models expose cells to the serum obtained from a participant at a particular moment in time, meaning the serum is simply a snapshot of time and this may not truly reflect the continuous changes observed *in vivo* across a period. Consequently, caution must be taken when interpreting results from studies using exercised serum or serum from individuals at varying degrees of training status *in vitro* unless the supply of serum is refreshed or the concentration of inflammatory markers of interest are administered in a way that replicates physiological changes after exercise.

Longitudinal studies can provide a clearer insight into the effects of long-term participation in physical activity and exercise on inflammatory markers. Hamer et al. [53] investigated the association between self-reported activity levels and inflammatory markers in 4289 men and women with a mean age of 49.2 years at starting point, over a 10-year period from the Whitehall II cohort study. They reported that physically active participants had lower IL-6 and CRP levels at baseline, and this remained stable over time. Those participants who reported an increase in physical activity from baseline also displayed lower levels of CRP and IL-6 at follow up. However, the levels of physical activity in this study were classified as ‘meeting physical activity guidelines’; at least 2.5 h of moderate to vigorous activity levels per week. These levels are vastly different to those of the trained ‘athletes’ in our study who reported at least 6.7 h of training per week. Another study investigated the effects of 9 months of endurance training on CRP levels in runners preparing for a marathon [48, 49, 54]. They found that after 9 months of endurance training, CRP levels decreased (*P* < 0.05). They also looked at CRP levels in untrained control participants and did not observe any differences in CRP levels after 9 months, further supporting the notion that long-term endurance training can exert an anti-inflammatory effect. These literature findings lend further credence the results of the present study whereby we observe that invasion of MDA-MB-231 cells cultured in the trained serum (both young and older) was lower when compared with that of age-matched control participants. Moreover, these findings align with another related study whereby it was observed that older participants who were sprint or endurance trained had lower levels of CRP than older control participants [41]

### Limitations

It should be noted that there is high variability in the reported effects of acute and chronic-exercise conditioned serum on *in vitro* models of cancer progression [55]. These discrepancies could be due to numerous factors, including the range of diverse populations used (i.e., older vs younger, diseased vs healthy, trained vs untrained, sedentary vs active), different cell lines used and the conditions in which they are cultured, inaccuracy stemming from self-reporting levels of exercise [56], as well as different methodologies used. Intensity [57, 58], duration [59–61] and mode of exercise [62] is also known to have an important impact on the serologic response seen during and after exercise, and it can therefore be speculated that the type, duration and intensity of exercise will differentially affect the preventative effects of exercised-serum against cancer.

Additional limitations to the current study include the relatively small sample size used, which may have contributed to the non-significant findings that were found. The media was changed every 24 h in this study therefore cytokine levels may well have depleted completely before being replenished, which does not truly represent physiological events observed *in vivo*. Furthermore, differences in BMI and body fat % between groups may also have contributed to the effects seen. Lower levels of body fat are known to cause changes in plasma profiles, including shifts in key proteins/inflammatory cytokines involved in tumourigenesis [63]. Participants in this study self-reported levels of physical activity, which has the potential for numerous measurement errors [56] and therefore must be treated with caution. Furthermore, pooling serum from male and female participants could potentially mask the effects from differences in sex hormones in the blood, however, this may be minimal since it is known that physical activity only has a modest effect on altering sex hormones levels in men [64] and women [65]. Finally, there was no data available as to when the participants last conducted physical activity before their blood samples were taken, and therefore it is uncertain whether the effects observed are solely due to training status and not due to a possible acute response to exercise instead.

### Future research

Future studies investigating whether chronically trained individuals (defined herein as 11 + years of regular training) who complete an acute bout of exercise have the same serological responses compared to untrained individuals, and whether sera from these individuals would attenuate breast cancer cell growth would be of interest. Study design is paramount to be able to determine these effects. It is still unclear whether the association between exercise and/or physical activity and inflammatory markers is independent of levels of adiposity, diet and several other possible confounding variables [48, 49, 66]. Future research focused on study design is required to ensure confounding factors such as body composition and diet are eliminated so the effects of long-term exercise training alone can be determined. The following study parameters may serve as a prudent starting point: trained vs untrained individuals completing an acute bout of exercise where diet, adiposity, time (to account for circadian fluctuations in serum profile) and where recent exercise bouts are accounted/controlled for.

## Conclusion

To the best of our knowledge, this is the first study that investigates the long-term effect of exercise training type in two different populations (younger vs older), on the invasion of breast cancer cells and mesenchymal stem cells *in vitro.* Invasion of MDA-MB-231 breast cancer cells was significantly lower in cells cultured in serum from younger, inactive participants compared with older, inactive participants, suggesting age protects against cancer invasiveness *in vitro*. Critically, no significant difference was observed between the invasion of MDA-MB-231 breast cancer cells cultured in serum from older, long-term endurance runners and younger, long-term endurance runners. These findings suggest a potentially protective effect of long-term endurance running against the naturally observed increases in invasion observed with age in the cells cultured with control participants’ serum, and favour endurance running over sprinting for these effects. Further research focusing on study design is required to truly understand the effects of long-term exercise training on the invasion of breast cancer cells in vitro, and whether chronic endurance training could have a protective effect against the “inflammageing” concept observed with age.

## Data Availability

The datasets generated during and/or analysed during the current study are available from the corresponding author on reasonable request.
