# Publisher Correction: The protective effect of endurance running against the pro-invasive effects of ageing in breast cancer cells and mesenchymal stem cells in vitro

**DOI:** 10.1007/s44164-024-00069-0

**Published:** 2024-04-26

**Authors:** Marie-Juliet Brown, Matt Nickels, Elizabeth C. Akam, Mhairi A. Morris

**Affiliations:** School of Sport, Exercise and Health Sciences, Loughborough University, Towers Way, Loughborough, LE11 3TU UK

**Publisher Correction: In vitro models (2023) 2:263-280**

https://doi.org/ 10.1007/s44164-023-00055-y

In this article the incorrect images were assigned to Figs. 2-6:

Image of Fig. 2 was incorrectly assigned to Fig. 4.

**Fig. 2:**
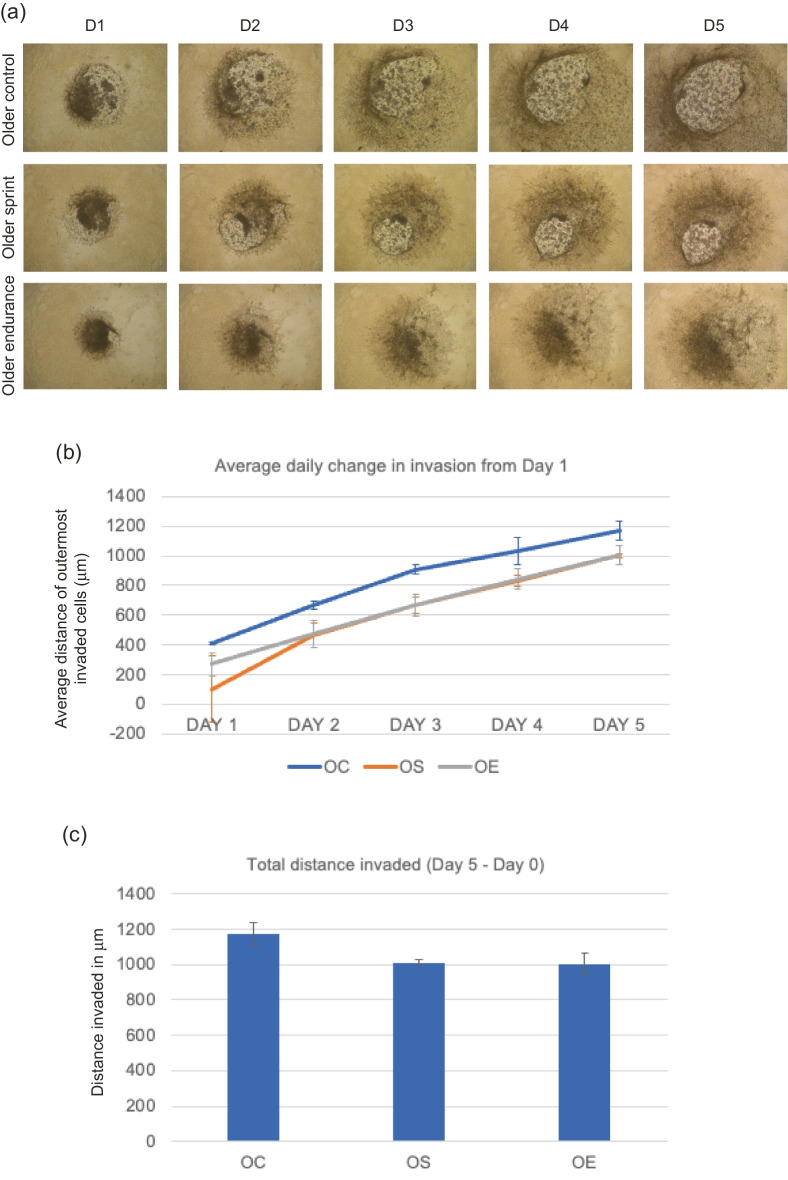
**a** Representative images of MDA-MB-231 spheroid invasion in a BME/rat collagen Type I matrix from Day 1 to Day 5 in OLDER control, sprint and endurance exercised serum; **b** average distance (mm) of outermost invaded cells was measured using the multi-point tool on ImageJ, and data were analysed using one-way ANOVAs and a repeated measures generalized linear model to determine main effects; **(c)** Total distance invaded was measured by subtracting the distance invaded by day 1 from the distance invaded by day 5. No significant main effect of condition was observed (P = 0.219) and the reduction in total invasion in spheroids cultured in older serum was also not significant (P = 0.139). Data representative of biological triplicates with standard error bars presented. Abbreviations: OC = older control; OE = older endurance; OS = older sprint

Image of Fig. 3 was incorrectly assigned to Fig. 5.

**Fig. 3:**
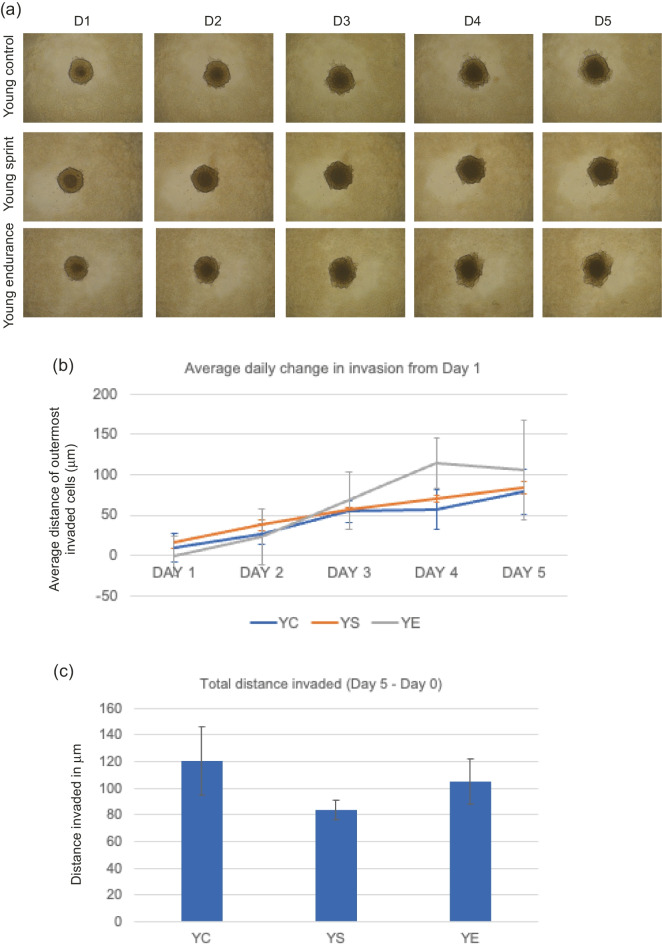
**a** Representative images of T47D spheroid invasion in a BME/ rat collagen Type I matrix from Day 1 to Day 5 in YOUNG control, sprint and endurance exercised serum; **b** average distance (mm) of outermost invaded cells was measured using the multi-point tool on ImageJ, and data were analysed using one-way ANOVAs and a repeated measures generalized linear model to determine main effects; **(c)** Total distance invaded was measured by subtracting the distance invaded by day 1 from the distance invaded by day 5. No significant main effect of condition was observed (P = 0.878) and the reduction in total invasion in spheroids cultured in young serum was also not significant (P = 0.801). Data representative of biological triplicates with standard error bars presented. Abbreviations: YC = young control; YE = young endurance; YS = young sprint

Image of Fig. 4 was incorrectly assigned to Fig. 6.

**Fig. 4:**
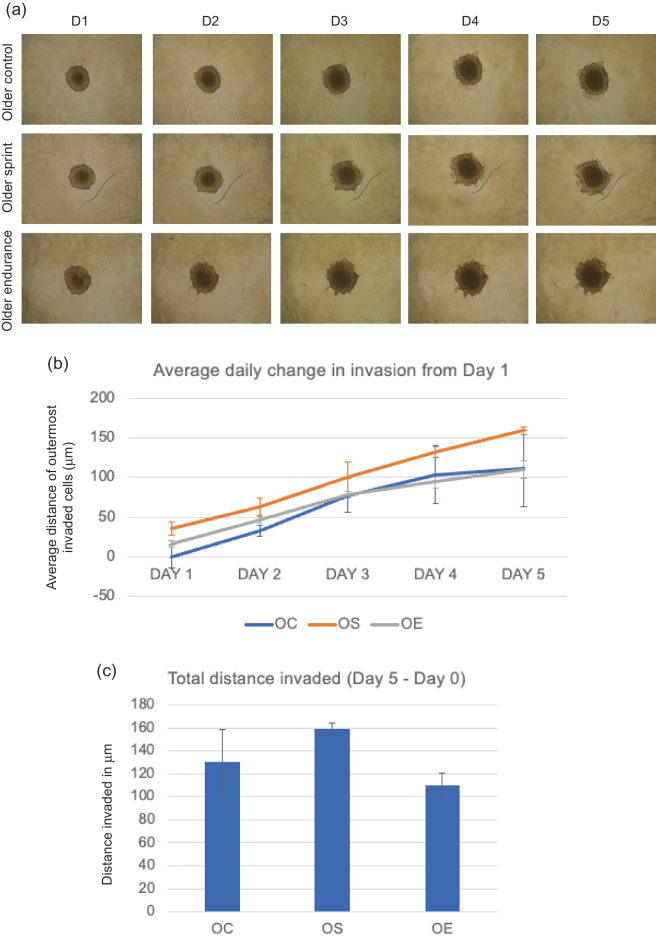
**a** Representative images of T47D spheroid invasion in a BME/ rat collagen Type I matrix from Day 1 to Day 5 in OLDER control, sprint and endurance exercised serum; **b** average distance (mm) of outermost invaded cells was measured using the multi-point tool on ImageJ, and data were analysed using one-way ANOVAs and a repeated measures generalized linear model to determine main effects; **(c)** Total distance invaded was measured by subtracting the distance invaded by day 1 from the distance invaded by day 5. No significant main effect of condition was observed (P = 0.596) and the reduction in total invasion in spheroids cultured in older serum was also not significant (P = 0.706). Data representative of biological triplicates with standard error bars presented. Abbreviations: OC = older control; OE = older endurance; OS = older sprint

Image of Fig. 5 was incorrectly assigned to Fig. 2.

**Fig. 5:**
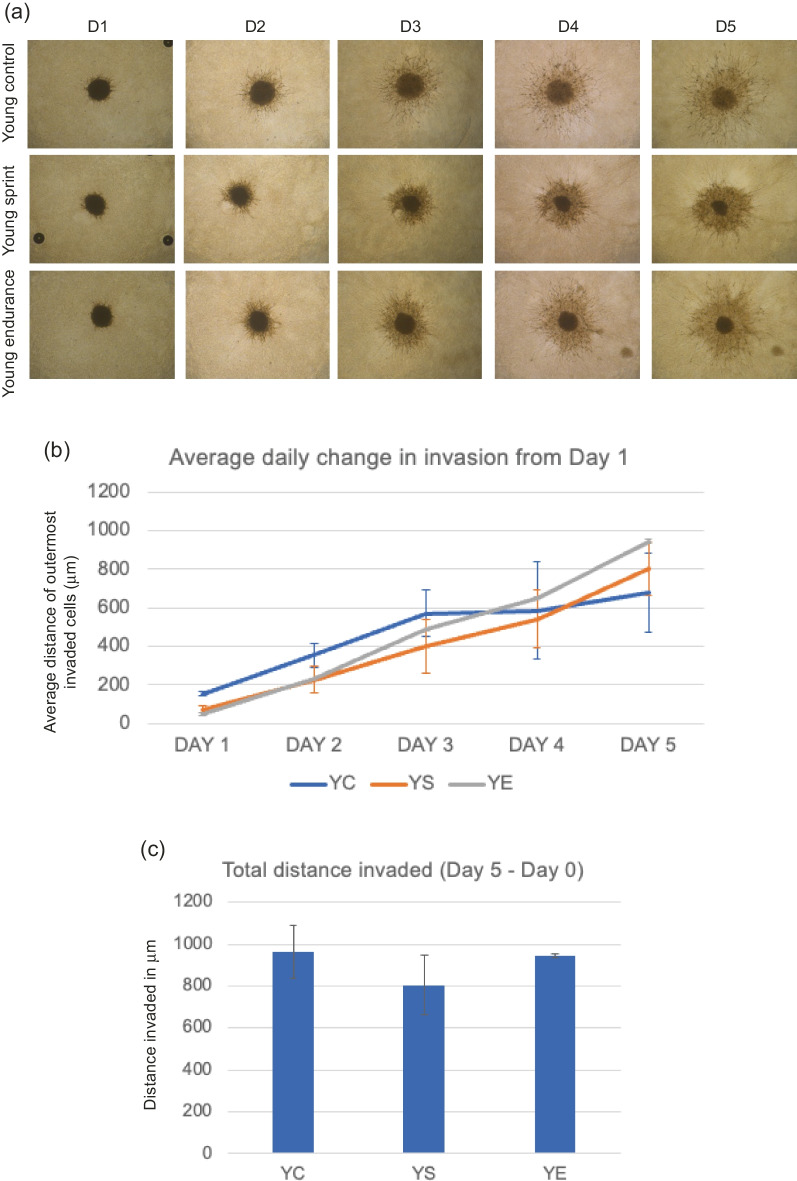
**a** Representative images of hBM-MSC spheroid invasion in a BME/rat collagen Type I matrix from Day 1 to Day 5 in YOUNG control, sprint and endurance exercised serum; **b** average distance (mm) of outermost invaded cells was measured using the multi-point tool on ImageJ, and data were analysed using one-way ANOVAs and a repeated measures generalized linear model to determine main effects; **c** Total distance invaded was measured by subtracting the distance invaded by day 1 from the distance invaded by day 5. The distance invaded by spheroids cultured in YC serum on Day 1 was significantly greater than those cultured in YS serum (P = 0.018) and YE serum (P = 0.011). No significant main effect of condition was observed (P = 0.892) and the reduction in total invasion in spheroids cultured in young serum was also not significant (P = 0.635). Data representative of biological triplicates with standard error bars presented. Abbreviations: YC = young control; YE = young endurance; YS = young sprint

Image of Fig. 6 was incorrectly assigned to Fig. 3.

**Fig. 6:**
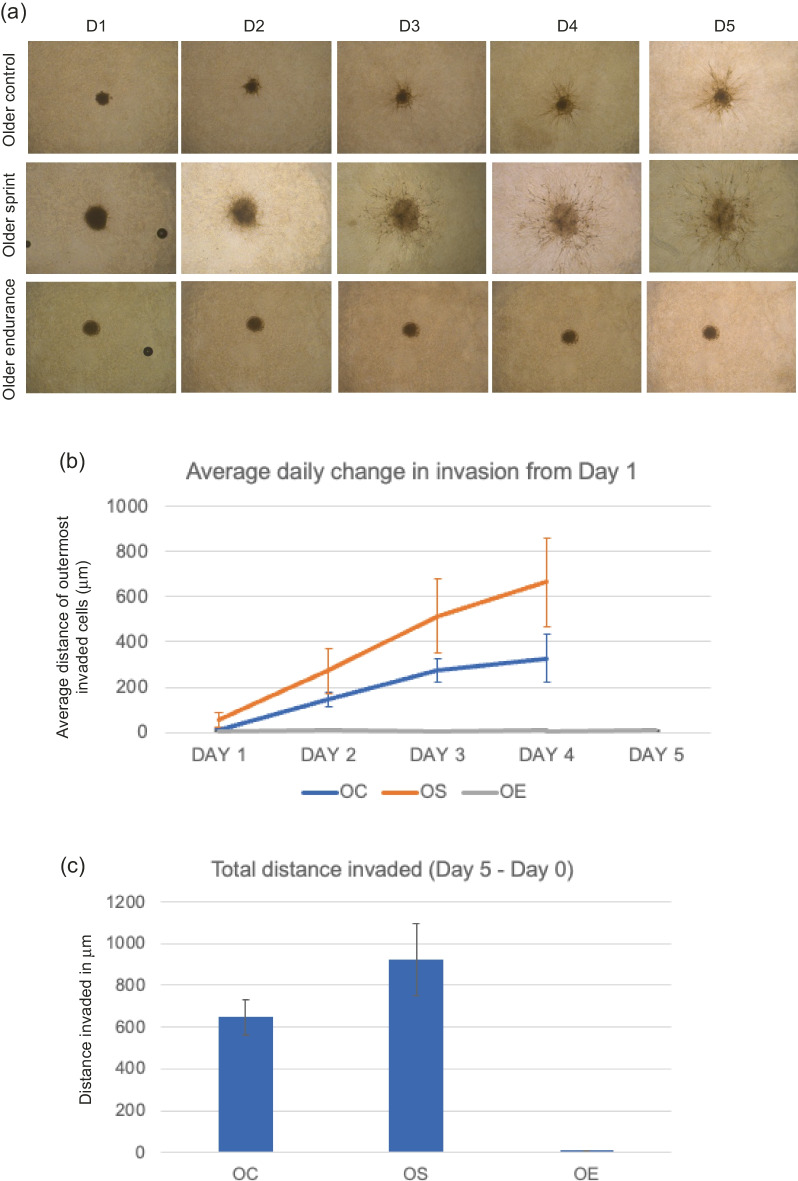
**a** Representative images of hBM-MSC spheroid invasion in a BME/rat collagen Type I matrix from Day 1 to Day 5 in OLDER control, sprint and endurance exercised serum; **b** average distance (mm) of outermost invaded cells was measured using the multipoint tool on ImageJ, and data were analysed using one-way ANOVAs and a repeated measures generalized linear model to determine main effects; **(c)** Total distance invaded was measured by subtracting the distance invaded by day 1 from the distance invaded by day 5. A significant main effect of condition was observed (P = 0.020) and the reduction in total invasion in spheroids cultured in older serum was also not significant (P = 0.003). Data representative of biological triplicates with standard error bars presented. Abbreviations: OC = older control; OE = older endurance; OS = older sprint

The correct order of figures is as listed below.

The original article has been corrected.

